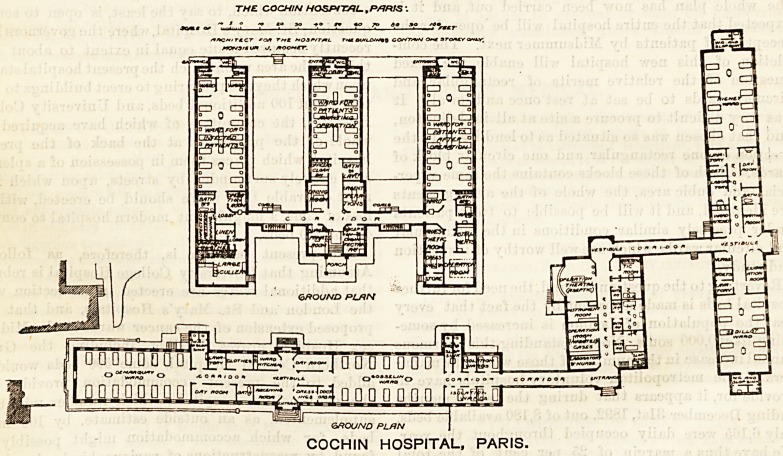# The Cochin Hospital, Paris

**Published:** 1894-02-03

**Authors:** 


### HOSPITAL CONSTRUCTION.

### THE COCHIN HOSPITAL, PARIS.

The Cochin Hospital was founded in 1780 by the benevolent Abbé whose name it now bears, who was Curé of the parish of St. Jacques du-Haut-pas. "The modesty of the founder," says M. Husson, "had given to the hospice no other name than that of the Hospice de Saint Jacques du-Haut-pas;" and by that title it is referred to in Tenon's "Work on the Paris Hospitals." Originally it contained but sixteen "petits lits," (*i.e.* single beds) for men, and eighteen for women. Some of the patients paid a "pension" of 450 livres, others 500 livres; those paying the larger sum had separate rooms, the others were mixed with the patients who were treated gratuitously.

In Husson's time (1862) the number of patients had increased to 119, of whom 50 were medical and 51 surgical cases. The remainder is accounted for by eight beds for lying-in women and ten cots.

In the latest edition of Oppert's "Work on Hospitals" the Cochin is stated to be one of the smallest of the Paris hospitals, and the total number of beds is given as 249, of which 44 were cribs.

At the present time the accommodation is about 400 beds, including a "baraque" ward for infectious cases.

The hospital was enlarged in 1893 by the erection of two groups of buildings, the plans of which, through the courtesy of the architect, M. J. Rochet, we are enabled to publish to-day.

1. Pavillon Lister. This group consists of a small building, containing an operation theatre and its adjuncts, and which bears above its entrance portal the words "Pavillon Lister;" a graceful dedication to our own great surgeon, of a new home of antiseptic surgery; and of two pavilions of wards, one for men, the other for women. The buildings are all of one storey only, and the wards are connected with the central block by covered ways, freely ventilated at each side.

The operation room building contains a large theatre in which are a couple of rows of staging for students, entered from the outside. The greatest care has been exercised here to preserve aseptic conditions as far as possible. The floor is of terrazzo, the walls of polished cement, and all the angles are carefully rounded. The sinks are of glass. In the wall at one side is a recessed cupboard, with doors of iron, which forms a hot closet for linen, &c. Immediately adjoining the operation theatre is the instrument room. On the opposite side of the corridor is a small room in which anæsthetics are administered; and in the recess outside this room the operation table is kept when not in use. The table is constructed of iron framework, nickel-plated throughout, and is a really beautiful piece of mechanism. A smaller operation room adjoins the instrument room, and next to it is a room for the nurse in charge of this department. The four small rooms on the right of the corridor are isolation wards for patients after operation. Each of these rooms has a small and rather dark lobby, the use of which is not very clear. The one weak point in the plan is the position of the w.c. and sinks, which are inside the building and open out of the main corridor. This mistake is all the more remarkable as the w.c's. attached to the ward pavilions are all separated by ventilated lobbies. The pavilion for male patients contains one large ward of 16 beds, one of 14 beds, three smaller wards of two beds each, and one of a single bed. In the centre is an entrance hall, with a door on each side giving acccess to the garden. Here also are the receiving room for patients, with surgeon's room adjoining, two large day rooms for convalescent patients,

COCHIN HOSPITAL, PARIS.

a ward kitchen, clothes store, lavatory, and bath room. The female block is arranged very similarly, but contains a total of 22 patients instead of 37, and has no small wards.

The walls of this group of buildings are of brick, and all have cellars under, which are used as store-rooms. The roofs are of iron framework, covered with tiles. The floors of all the rooms are of impermeable ceramic tiles. The wards are warmed by double fire-places, and the vestibules and lobbies by calorifers. The cubic space per bed is about 1,600 feet, and the cost per bed is stated to have been about £214.

2. Pavillon Pasteur. This group of buildings, which bears the name of the great French savant, is wholly for women, the corresponding service for male patients being in the old part of the hospital. It consists of three wings planned in the form of an E, with the addition of three small projections on the left of the upstroke. In the centre building is the entrance, close to which is a room for the reception and disinfection of patients, and the surgeon's private room. Immediately beyond these rooms the corridor leads right and left to the side wings. Passing this corridor and going towards the central ward are the following: Room for urgent operations, dining room for patients, cloak room for medical officers, bath room, and lavatory. The central ward is for patients awaiting operation, and contains 10 beds; at the extreme end is a room for soiled linen, a coal store, w.c., and entrance.

The left-hand wing contains a ward for 12 beds, two small single-bed wards, and the usual ward offices, including a dining room for patients.

The right-hand pavilion contains a large ward for 14 beds, two small single-bed wards, an operation room, instrument room, room for the administration of anæsthetics, and the usual ward offices.

The construction of these buildings is entirely of wooden framework, on the "système Pourbla," with a filling of bricks made of slag from blast furnaces. The roofs are covered with tiles. The floors of the wards are of parquet of pitch pine, those of the accessory rooms of mastic cement, and that of the operation room of ceramic tiles. All the buildings are raised about 5 feet. above the ground. The wards are warmed by double ventilating grates, and the operation room by a calorifer. The cubic space per bed is about 1,339 feet., and the cost per bed is stated to have been about £168.

## Figures and Tables

**Figure f1:**